# Supplementary material for: Multi‐dataset identification of innovative feature genes and molecular mechanisms in keratoconus
Source: J Cell Mol Med. 2024 Sep 19;28(18):e70079. doi: 10.1111/jcmm.70079 (PMC11412914; doi:10.1111/jcmm.70079)
Supplement: Supplementary file 2 — Material S2. [file JCMM-28-e70079-s002.zip › 7.GSEA analysis/2.ATOH7/KEGG_PURINE_METABOLISM.html]

Details for gene set KEGG\_PURINE\_METABOLISM[GSEA]

|  || Dataset | ATOH7.group.cls#h\_versus\_l |
| Phenotype | group.cls#h\_versus\_l |
| Upregulated in class | h |
| GeneSet | KEGG\_PURINE\_METABOLISM |
| Enrichment Score (ES) | 0.30916938 |
| Normalized Enrichment Score (NES) | 1.3322769 |
| Nominal p-value | 0.13148148 |
| FDR q-value | 0.33797517 |
| FWER p-Value | 0.946 |
Table: GSEA Results Summary

  

Fig 1: Enrichment plot: KEGG\_PURINE\_METABOLISM      
 Profile of the Running ES Score & Positions of GeneSet Members on the Rank Ordered List

  

| SYMBOL | TITLE | RANK IN GENE LIST | RANK METRIC SCORE | RUNNING ES | CORE ENRICHMENT || 1 | PDE8B | na | 25 | 0.956 | 0.0501 | Yes |
| 2 | GMPR | na | 126 | 0.743 | 0.0867 | Yes |
| 3 | URAD | na | 302 | 0.624 | 0.1149 | Yes |
| 4 | PDE4B | na | 317 | 0.617 | 0.1473 | Yes |
| 5 | PDE1B | na | 538 | 0.537 | 0.1695 | Yes |
| 6 | NME4 | na | 1469 | 0.389 | 0.1633 | Yes |
| 7 | PDE1A | na | 1971 | 0.346 | 0.1672 | Yes |
| 8 | PFAS | na | 2254 | 0.326 | 0.1764 | Yes |
| 9 | RRM2 | na | 2497 | 0.310 | 0.1858 | Yes |
| 10 | PAPSS2 | na | 2587 | 0.304 | 0.1994 | Yes |
| 11 | AMPD2 | na | 2700 | 0.298 | 0.2120 | Yes |
| 12 | PDE5A | na | 2945 | 0.283 | 0.2200 | Yes |
| 13 | ADCY10 | na | 2975 | 0.282 | 0.2342 | Yes |
| 14 | PDE11A | na | 3073 | 0.277 | 0.2461 | Yes |
| 15 | NME7 | na | 4035 | 0.235 | 0.2308 | Yes |
| 16 | PRPS1L1 | na | 4168 | 0.230 | 0.2392 | Yes |
| 17 | PDE7B | na | 4282 | 0.226 | 0.2480 | Yes |
| 18 | AMPD1 | na | 4515 | 0.218 | 0.2529 | Yes |
| 19 | PDE2A | na | 4708 | 0.211 | 0.2585 | Yes |
| 20 | NT5M | na | 4728 | 0.210 | 0.2692 | Yes |
| 21 | PRPS2 | na | 4791 | 0.208 | 0.2784 | Yes |
| 22 | ADCY3 | na | 5082 | 0.198 | 0.2806 | Yes |
| 23 | PDE4A | na | 5301 | 0.192 | 0.2845 | Yes |
| 24 | ADPRM | na | 6065 | 0.169 | 0.2714 | Yes |
| 25 | ENPP1 | na | 6112 | 0.168 | 0.2790 | Yes |
| 26 | POLE2 | na | 6387 | 0.160 | 0.2796 | Yes |
| 27 | PRIM1 | na | 6630 | 0.153 | 0.2807 | Yes |
| 28 | HPRT1 | na | 6690 | 0.151 | 0.2871 | Yes |
| 29 | RRM2B | na | 6700 | 0.151 | 0.2949 | Yes |
| 30 | AK7 | na | 6790 | 0.149 | 0.3002 | Yes |
| 31 | POLA2 | na | 6919 | 0.145 | 0.3042 | Yes |
| 32 | ENTPD1 | na | 7011 | 0.143 | 0.3092 | Yes |
| 33 | PDE6D | na | 7296 | 0.136 | 0.3082 | No |
| 34 | POLR1E | na | 7944 | 0.120 | 0.2958 | No |
| 35 | PDE6B | na | 8091 | 0.117 | 0.2978 | No |
| 36 | PAICS | na | 8218 | 0.114 | 0.3003 | No |
| 37 | PDE10A | na | 8396 | 0.110 | 0.3010 | No |
| 38 | NME5 | na | 8422 | 0.110 | 0.3061 | No |
| 39 | POLR3F | na | 8779 | 0.103 | 0.3013 | No |
| 40 | NPR2 | na | 9313 | 0.093 | 0.2908 | No |
| 41 | DCK | na | 9351 | 0.093 | 0.2947 | No |
| 42 | POLR3D | na | 9611 | 0.088 | 0.2919 | No |
| 43 | PDE6G | na | 9947 | 0.082 | 0.2866 | No |
| 44 | NPR1 | na | 10142 | 0.079 | 0.2852 | No |
| 45 | ADK | na | 10263 | 0.077 | 0.2858 | No |
| 46 | ADCY8 | na | 10448 | 0.075 | 0.2844 | No |
| 47 | POLR1D | na | 10453 | 0.074 | 0.2883 | No |
| 48 | PKM | na | 10652 | 0.071 | 0.2863 | No |
| 49 | RRM1 | na | 10703 | 0.071 | 0.2886 | No |
| 50 | POLR2K | na | 10875 | 0.068 | 0.2873 | No |
| 51 | IMPDH2 | na | 11124 | 0.064 | 0.2835 | No |
| 52 | GUK1 | na | 11411 | 0.060 | 0.2785 | No |
| 53 | PDE8A | na | 11736 | 0.056 | 0.2721 | No |
| 54 | POLD3 | na | 11763 | 0.056 | 0.2743 | No |
| 55 | CANT1 | na | 11936 | 0.053 | 0.2722 | No |
| 56 | POLA1 | na | 12102 | 0.051 | 0.2701 | No |
| 57 | GMPS | na | 12108 | 0.051 | 0.2727 | No |
| 58 | POLR3GL | na | 12115 | 0.051 | 0.2752 | No |
| 59 | POLD2 | na | 12245 | 0.050 | 0.2741 | No |
| 60 | POLR3G | na | 12290 | 0.049 | 0.2754 | No |
| 61 | ENTPD3 | na | 12454 | 0.047 | 0.2732 | No |
| 62 | POLR2L | na | 12507 | 0.046 | 0.2742 | No |
| 63 | POLE3 | na | 12705 | 0.044 | 0.2708 | No |
| 64 | DGUOK | na | 12752 | 0.043 | 0.2717 | No |
| 65 | NME2 | na | 13527 | 0.035 | 0.2512 | No |
| 66 | POLR3K | na | 13715 | 0.033 | 0.2476 | No |
| 67 | NME1 | na | 13937 | 0.031 | 0.2428 | No |
| 68 | NUDT5 | na | 14033 | 0.030 | 0.2417 | No |
| 69 | POLR2E | na | 14079 | 0.030 | 0.2420 | No |
| 70 | PRPS1 | na | 14361 | 0.027 | 0.2353 | No |
| 71 | POLR2D | na | 14385 | 0.027 | 0.2361 | No |
| 72 | POLE4 | na | 14460 | 0.026 | 0.2353 | No |
| 73 | PRIM2 | na | 14572 | 0.025 | 0.2334 | No |
| 74 | AK1 | na | 14886 | 0.022 | 0.2255 | No |
| 75 | NUDT9 | na | 14922 | 0.022 | 0.2257 | No |
| 76 | POLR2J | na | 15173 | 0.020 | 0.2195 | No |
| 77 | POLR2G | na | 15364 | 0.018 | 0.2150 | No |
| 78 | NT5C3A | na | 15465 | 0.017 | 0.2130 | No |
| 79 | NME6 | na | 15514 | 0.017 | 0.2125 | No |
| 80 | FHIT | na | 15594 | 0.016 | 0.2111 | No |
| 81 | ATIC | na | 15696 | 0.015 | 0.2089 | No |
| 82 | PDE9A | na | 15769 | 0.015 | 0.2076 | No |
| 83 | POLR2B | na | 15837 | 0.014 | 0.2064 | No |
| 84 | GMPR2 | na | 15876 | 0.014 | 0.2061 | No |
| 85 | POLR2I | na | 15931 | 0.013 | 0.2052 | No |
| 86 | AMPD3 | na | 16050 | 0.013 | 0.2025 | No |
| 87 | GUCY2F | na | 16611 | 0.008 | 0.1867 | No |
| 88 | ENTPD5 | na | 16633 | 0.008 | 0.1865 | No |
| 89 | POLR2F | na | 16643 | 0.008 | 0.1867 | No |
| 90 | ENPP3 | na | 17063 | 0.004 | 0.1748 | No |
| 91 | POLR1B | na | 17066 | 0.004 | 0.1749 | No |
| 92 | PPAT | na | 17099 | 0.004 | 0.1742 | No |
| 93 | PNPT1 | na | 17363 | 0.001 | 0.1666 | No |
| 94 | AK2 | na | 17548 | -0.000 | 0.1613 | No |
| 95 | POLR2J3 | na | 17578 | -0.000 | 0.1605 | No |
| 96 | APRT | na | 17802 | -0.002 | 0.1542 | No |
| 97 | POLR1C | na | 18124 | -0.005 | 0.1452 | No |
| 98 | POLR3A | na | 18374 | -0.007 | 0.1383 | No |
| 99 | NME3 | na | 18442 | -0.008 | 0.1368 | No |
| 100 | POLR2A | na | 18527 | -0.009 | 0.1349 | No |
| 101 | ADSL | na | 18584 | -0.009 | 0.1337 | No |
| 102 | PNP | na | 18817 | -0.011 | 0.1276 | No |
| 103 | POLD4 | na | 19537 | -0.017 | 0.1078 | No |
| 104 | ADA | na | 19645 | -0.018 | 0.1057 | No |
| 105 | AK5 | na | 19725 | -0.019 | 0.1044 | No |
| 106 | POLR2C | na | 19762 | -0.019 | 0.1044 | No |
| 107 | ENTPD4 | na | 19915 | -0.021 | 0.1011 | No |
| 108 | PDE6H | na | 20420 | -0.026 | 0.0879 | No |
| 109 | POLD1 | na | 20660 | -0.028 | 0.0825 | No |
| 110 | XDH | na | 20671 | -0.028 | 0.0837 | No |
| 111 | ADCY9 | na | 20701 | -0.029 | 0.0844 | No |
| 112 | POLR3B | na | 20856 | -0.030 | 0.0816 | No |
| 113 | POLR2H | na | 20975 | -0.031 | 0.0798 | No |
| 114 | IMPDH1 | na | 21049 | -0.032 | 0.0794 | No |
| 115 | GUCY2D | na | 21205 | -0.034 | 0.0767 | No |
| 116 | GART | na | 21419 | -0.036 | 0.0725 | No |
| 117 | ALLC | na | 21440 | -0.036 | 0.0738 | No |
| 118 | POLR2J2 | na | 21612 | -0.038 | 0.0709 | No |
| 119 | ADCY2 | na | 21650 | -0.039 | 0.0719 | No |
| 120 | NT5C | na | 21754 | -0.040 | 0.0711 | No |
| 121 | PDE3A | na | 22259 | -0.046 | 0.0590 | No |
| 122 | NUDT2 | na | 22863 | -0.053 | 0.0443 | No |
| 123 | AK4 | na | 22919 | -0.054 | 0.0456 | No |
| 124 | PAPSS1 | na | 23406 | -0.061 | 0.0348 | No |
| 125 | NT5C1A | na | 23540 | -0.063 | 0.0342 | No |
| 126 | ITPA | na | 23953 | -0.069 | 0.0260 | No |
| 127 | ENTPD2 | na | 24099 | -0.071 | 0.0256 | No |
| 128 | POLE | na | 24130 | -0.071 | 0.0285 | No |
| 129 | PDE6A | na | 24358 | -0.075 | 0.0259 | No |
| 130 | POLR3H | na | 24482 | -0.077 | 0.0265 | No |
| 131 | GDA | na | 24534 | -0.078 | 0.0291 | No |
| 132 | ADCY5 | na | 25230 | -0.089 | 0.0138 | No |
| 133 | ADCY6 | na | 25322 | -0.091 | 0.0160 | No |
| 134 | PDE1C | na | 26063 | -0.105 | 0.0002 | No |
| 135 | POLR1A | na | 26166 | -0.108 | 0.0029 | No |
| 136 | ENTPD6 | na | 26341 | -0.111 | 0.0038 | No |
| 137 | ADCY7 | na | 26708 | -0.119 | -0.0004 | No |
| 138 | ADCY4 | na | 27391 | -0.136 | -0.0129 | No |
| 139 | PDE4D | na | 29009 | -0.179 | -0.0502 | No |
| 140 | GUCY2C | na | 29225 | -0.186 | -0.0465 | No |
| 141 | PDE4C | na | 29331 | -0.189 | -0.0395 | No |
| 142 | NT5C2 | na | 29404 | -0.191 | -0.0314 | No |
| 143 | PKLR | na | 30222 | -0.219 | -0.0434 | No |
| 144 | ADCY1 | na | 30908 | -0.244 | -0.0502 | No |
| 145 | PDE3B | na | 32353 | -0.316 | -0.0752 | No |
| 146 | PDE6C | na | 32484 | -0.326 | -0.0616 | No |
| 147 | NT5C1B | na | 33398 | -0.404 | -0.0665 | No |
| 148 | ENTPD8 | na | 33525 | -0.418 | -0.0479 | No |
| 149 | GUCY1A2 | na | 33901 | -0.477 | -0.0334 | No |
| 150 | PDE7A | na | 34147 | -0.530 | -0.0123 | No |
| 151 | NT5E | na | 34221 | -0.547 | 0.0147 | No |
Table: GSEA details [plain text format]

  

Fig 2: KEGG\_PURINE\_METABOLISM      
 Blue-Pink O' Gram in the Space of the Analyzed GeneSet

  

Fig 3: KEGG\_PURINE\_METABOLISM: Random ES distribution      
 Gene set null distribution of ES for **KEGG\_PURINE\_METABOLISM**
